# Postoperative hypothalamic-pituitary dysfunction and long-term hormone replacement in patients with childhood-onset craniopharyngioma

**DOI:** 10.3389/fendo.2023.1241145

**Published:** 2023-11-06

**Authors:** Yuqi Miao, Kaiyu Fan, Xiaojiao Peng, Si Li, Jiahui Chen, Robin N. Bai, Yu Wei, Yaxian Deng, Chengsong Zhao, Qingfeng Wu, Ming Ge, Jian Gong, Di Wu

**Affiliations:** ^1^ Department of Endocrinology, Genetics and Metabolism, Beijing Children’s Hospital, Capital Medical University, Beijing, China; ^2^ Department of Pediatric Neurosurgery, Beijing Tiantan Hospital, Capital Medical University, Beijing, China; ^3^ Department of Neurosurgery, Beijing Children’s Hospital, Capital Medical University, Beijing, China; ^4^ State Key Laboratory of Molecular Development Biology, Institute of Genetics and Developmental Biology, Chinese Academy of Sciences, Beijing, China; ^5^ Department of Microbiology And Molecular Cell Biology, Eastern Virginia Medical School, Norfolk, VA, United States; ^6^ Department of Pediatric, Beijing Tiantan Hospital, Capital Medical University, Beijing, China; ^7^ Beijing Children’s Hospital, Capital Medical University, Beijing, China; ^8^ Beijing Key Laboratory for Genetics of Birth Defects, Beijing, China

**Keywords:** craniopharyngioma, hypothalamic-pituitary axis, hormone replacement therapy, pediatric patients, long-term follow-up

## Abstract

**Objective:**

Hypothalamic-pituitary axis dysfunction is a common complication in post-operative craniopharyngioma(CP) patients, and it greatly impacts the long-term quality of life of such patients. To better understand the effects of postoperative hypothalamic-pituitary dysfunction and long-term hormone replacement therapy in patients with childhood CP, we assessed approximately 200 patients with childhood-onset CP postoperatively.

**Methods:**

Clinical details of patients with childhood-onset CP who underwent sellar tumor resection in Beijing Children’s Hospital and Beijing Tiantan Hospital from 2018 to 2019 were retrieved retrospectively. The participants were followed up to assess the effects of post-operative long-term hormone replacement therapy and assess the tumor recurrence rate.

**Results:**

The median age of admission was 8.1 (1.8, 14.3) years. Headache (45.5%), visual impairment (39.5%), and nausea (33.0%) were the most common clinical manifestations. ACP accounted for 95% of all CP cases. The incidence of central adrenal insufficiency and central hypothyroidism within the first week after surgery was 56.2% and 70.3%, respectively. At the same time 85.5% of the patients required at least one dose of desmopressin to control urine output. Total survival and tumor recurrence rates were 98.6% and 26.1%, respectively, with a median follow-up time of 29.7 (19.0, 40.3) months. During the follow-up period, 28.1% patients met the diagnostic criteria for short stature, while 54.4% fit the criteria for obesity. In addition, 94.4% of the patients were taking at least one kind of hormone substitution, and 74.7% were taking three or more. The prevalence of levothyroxine, glucocorticoid, desmopressin, and growth hormone replacement therapy was 87.3%, 77.5%, 78.9% and 31.0%, respectively. The proportion of patients treated with the substitutive combination of levothyroxine, hydrocortisone, and desmopressin was 54.9%.

**Conclusion:**

This study is a large-sample systematic postoperative endocrine function evaluation of patients with childhood-onset CP. Due to the high prevalence of post-operative hypothalamic-pituitary dysfunction, patients with CP usually require long-term multiple hormone substitution therapy. Individualized management and accurate hormone replacement dosage for postoperative childhood-onset CP patients are of great importance.

## 1 Introduction

Craniopharyngiomas (CPs) are benign tumors located mainly in the sellar region. There are two pathological types: adamantinomatous craniopharyngioma (ACP) and papillary craniopharyngioma (PCP) (1). Almost all childhood-onset CP patients present with ACP,while PCP mostly occurs exclusively in adulthood, from ages 45 to 60 years (2). The histological and genetic characteristics of ACP and PCP are also vastly different (3–5). Currently, the first-line treatment for childhood onset CPs is surgical resection while the preferred treatment in adults is surgical resection with adjuvant radiotherapy or radiotherapy alone (6).

The primary goal of surgical ablation is complete tumor removal with protection of the structure and function of the hypothalamic-pituitary axis. However. CPs occur adjacent to the hypothalamus-pituitary axis, optic chiasm, and other functional structures. Tumor invasion or extrusion and surgical or radiotherapeutic movements may damage these surrounding tissues and cause various complications (7).The 5-year and 10-year overall survival rates of pediatric patients with CP after surgery are already at a high level of 83–96% and 65–100%, respectively (8). Long-term monitoring of endocrine function and accurate endocrine hormone replacement therapy that meets the growth and development needs of patients are very important to improving their long-term prognosis. At present, there is still a lack of large-scale studies on the post-operative status of patients with childhood-onset CP in China, which explains the lack of a systematic and standardized endocrine management for most of these patients. Therefore, bettering our understanding of long-term hypothalamus-pituitary axis dysfunction and hormone replacement therapy is vital for creating a more rational and accurate management for postoperative CP patients. In this study, we retrospectively collected detailed clinical data and subsequently followed up on children with CP who underwent surgical resection in Beijing Children’s Hospital and Beijing Tiantan Hospital from 2018 to 2019.

## 2 Materials and methods

### 2.1 Participants

We retrospectively analyzed the clinical data of patients who underwent sellar tumor resection at the Neurosurgery Department of Beijing Children’s Hospital and Beijing Tiantan Hospital from January 2018 to December 2019. The histological diagnosis was confirmed as CP, including ACP and PCP. A total of 210 people met the inclusion criteria, all of them were below 18 years old at admission. Patients with other malignancies, renal failure, hormone deficiencies unrelated with CP and/or it’s treatment, and missing detailed pre-/postoperative hospitalization records were excluded. Those who died during the perioperative period were also excluded from this study. Finally a total of 200 patients with CP were included in this study.

### 2.2 Data collection

Clinical data, including basic information (age, sex, clinical manifestations at admission, date of surgery, surgical approach, and histological diagnosis of the tumor), endocrine status before and 1 week after the surgery (thyroid function, adrenocortical function, gonadal function, blood sodium, blood and urine osmotic pressure, height, and weight) were retrospectively reviewed. Data on tumor recurrence, complications, and hormone replacement therapy were collected at follow-up. The study was approved by Medical Ethics Committee of Beijing Children’s Hospital (2020-k-188).

### 2.3 Endocrine assessment

Central adrenal insufficiency: Normal or decreased adrenocorticotropic hormone (ACTH) with cortisol levels of <30 μg/L was diagnosed as central adrenal insufficiency. Cortisol levels between 30 and 150 μg/L with serum cortisol levels of <180 μg/L at 30 or 60 min during an ACTH stimulation test also indicated central adrenal insufficiency.

Central hypothyroidism: This was diagnosed as a serum thyroid stimulating hormone (TSH) level that is significantly lower than the reference range, with normal or low levels of free thyroxine.

Short stature: This was diagnosed as height below the 3rd percentile (-1.88 standard deviation) of individuals with the same age and sex.

Central diabetes insipidus: This was diagnosed as urine volume >3000 ml/(m^2^· d) with a negative urine glucose test and urine/blood osmotic concentration of <2. Once the histological diagnosis is confirmed as CP, water deprivation or desmopressin injection tests are no longer needed.

Obesity: This was diagnosed as body max index (BMI) that exceeds the 95th percentile for children of the same age and sex.

### 2.4 Statistical analysis

The data were analyzed using SPSS Statistics version 23.0 (IBM). Normally distributed continuous data are expressed as means and standard deviations. Non-normally distributed continuous data are expressed as medians and interquartile ranges. Kruskal Wallis test was used to compare the difference in the number of cases among different age stages. Differences between binary outcomes were compared using Pearson’s chi-square or Fisher’s exact test. A P-value less than 0.05 was considered statistically significant.

## 3 Results

### 3.1 Basic information of the included participants

**Figure 1:**
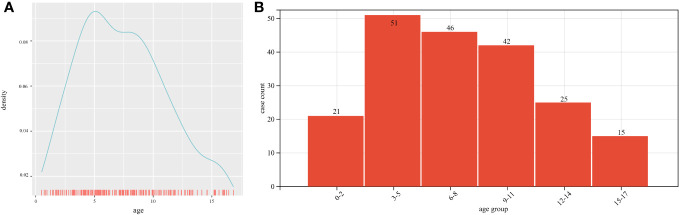
Age distribution: **(A)** Age distribution and probability density curve of patients at admission; **(B)** Number of cases according to age groups.

The median age at surgery was 8.1(1.8, 14.3) years. Age distribution and a probability density curve are shown in **Figure 1A**. Patients were divided into six groups according to their age. There was a modest, although not significant, difference in the number of cases among the groups (p=0.057) (**Figure 1B**). Regarding gender, 130 patients (65.0%) were male, and 70 patients (35.0%) were female. A total of 162 patients (81.0%) underwent first-time tumor resection, and 38 patients (19.0%) had undergone a previous tumor resection before the recent surgery.

Headache (45.5%), visual impairment (including decreased visual acuity or visual field defects)(39.5%), and nausea (33.0%) were the most common clinical manifestations. In addition, some patients presented with symptoms of hypothalamic-pituitary axis dysfunction, such as diabetes insipidus (14.5%). On admission, 38.0% and 25.2% of the patients met the diagnosis standard for obesity and short stature, respectively. In addition, nine cases (4.5%) did not present with a specific discomfort. Tumors were incidentally identified during brain imaging due to trauma or other reasons. A total of 17 cases (8.5%) underwent the transsphenoidal endoscopic endonasal approach, and 183 cases (91.5%) underwent transcranial resection of the tumors. There were 3 (1.5%) PCP cases and 190 (95%) ACP cases. The basic information of the participants is shown in **Table 1**.

**Table 1:** Basic information of the patients.

		Case	Total	N%
Age	0-2	21	200	10.5%
	3-5	51		25.5%
	6-8	46		23.0%
	9-11	42		21.0%
	12-14	25		12.5%
	15-17	15		7.5%
Sex	Male	130	200	65.0%
	Female	70		35.0%
Primary & Recurrence	Primary	162	200	81.0%
	Recurrence	38		19.0%
Primary Manifestation	Headache	91	200	45.5%
	Dizzy	20		10.0%
	Nausea	66		33.0%
	Visual defect	79		39.5%
	Polyuria	29		14.5%
	Convulsion	8		4.0%
	Lack of strength	12		6.0%
	Short stature	23	91	25.2%
	Obesity	76	200	38.0%
	No manifestation	9		4.5%
Surgical Approach	Transcranial tumor resection	183	200	91.5%
	Endoscopic endonasal transsphenoidal	17		8.5%
Histologic Diagnosis	ACP	190	200	95.0%
	PCP	3		1.5%
	undefined	7		3.5%

### 3.2 Perioperative endocrine hormone deficiency

Hypothalamic-pituitary axis function was evaluated before and 1 week after surgery. Thyroid function, adrenocortical function, growth hormone secretion, and antidiuretic hormone secretion were also assessed. The overall incidence of preoperative central adrenal insufficiency was 28.8%, among which incidence in patients with primary tumors was 16.2%. The overall incidence of preoperative central hypothyroidism was 16.3%, and in patients with primary tumors, the incidence was 5.6%. The incidence of central diabetes insipidus in patients with primary tumors was 11.8%. The rate of central adrenal insufficiency one week after surgery was 56.2%. The incidence of central hypothyroidism reached 70.3%. At least one dose of desmopressin was administered in 85.5% of the patients to control their urine output.

### 3.3 Long-term follow-up and hormone replacement therapy

**Figure 2:**
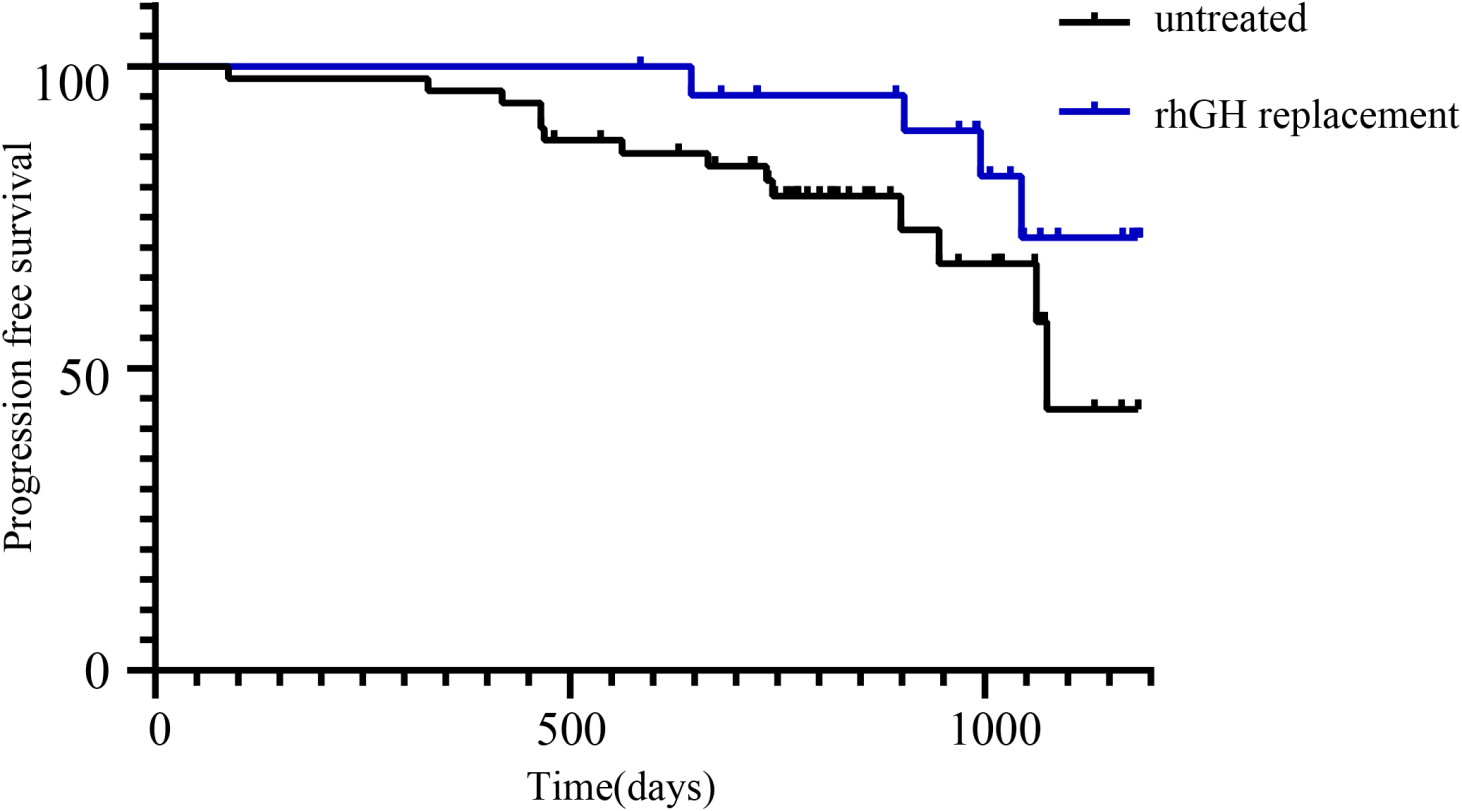
Kaplan-Meier curves of recurrence-free survival in patients treated with or without rhGH therapy. No significant difference was found between the two groups.

A total of 72 patients were followed up, with a median follow-up duration of 29.7 (19.0, 40.3) months. At the time of follow-up, 71 patients (98.6%) were alive. The major complaints reported by the 71 patients included headache, visual defects, polyuria, delayed puberty, short stature, and obesity. Current hormone substitution and tumor recurrence were also recorded (**Table 2**). Among the patients who regularly attended post-operative clinics, 18 (26.1%) had tumor recurrence, and 51 (73.9%) had no tumor recurrence. The mean duration before recurrence diagnosis was 23.2 ± 9.6 months. The patients were grouped based on the type of surgery received, primary or recurrent tumors, and the application or non-application of recombinant human growth hormone (rhGH). Patients who underwent the endoscopic endonasal transsphenoidal approach had a higher recurrence rate than those who received craniotomy, even though the difference was not statistically significant (22.6% vs. 57.1%, p=0.07). The recurrence rates between the other groups were also not statistically significant (**Table 3**). Kaplan Meier curves for progression-free survival showed no significant difference between patients who received and did not receive rhGH (**Figure 2**).

**Table 2:** Participant information at follow-up.

		Case	Total	N%
Alive or Death	Alive	71	72	98.6%
	Death	1		1.4%
Tumor Recurrence	Recurrence	18	69	26.1%
	No-recurrence	51		73.9%
Regular Review in Endocrinology Clinic	Yes	62	71	87.3%
	No	9		12.7%
Clinical Manifestation	Headache	5	71	7.0%
	Visual Defect	24		33.8%
	Polyuria	35		49.3%
	Short stature	16	57	28.1%
	Obesity	31		54.4%

**Table 3:** Analysis of factors related to postoperative tumor recurrence.

		Recurrence	Non-Recurrence	P Value
Surgical Approach	Craniotomy	14 (22.6%)	48 (77.4%)	p=0.07
	Endoscopic endonasal transsphenoidal	4 (57.1%)	3 (42.9%)	
Primary or Recurrence Tumor	Primary	16 (25.0%)	48 (75.0%)	p=0.60
	Recurrence	2 (40.0%)	3 (60.0%)	
rhGH Therapy	Yes	4 (18.2%)	18 (81.8%)	p=0.31
	No	14 (29.8%)	33 (70.2%)	

rhGH, recombinant human growth hormone.

**Figure 3:**
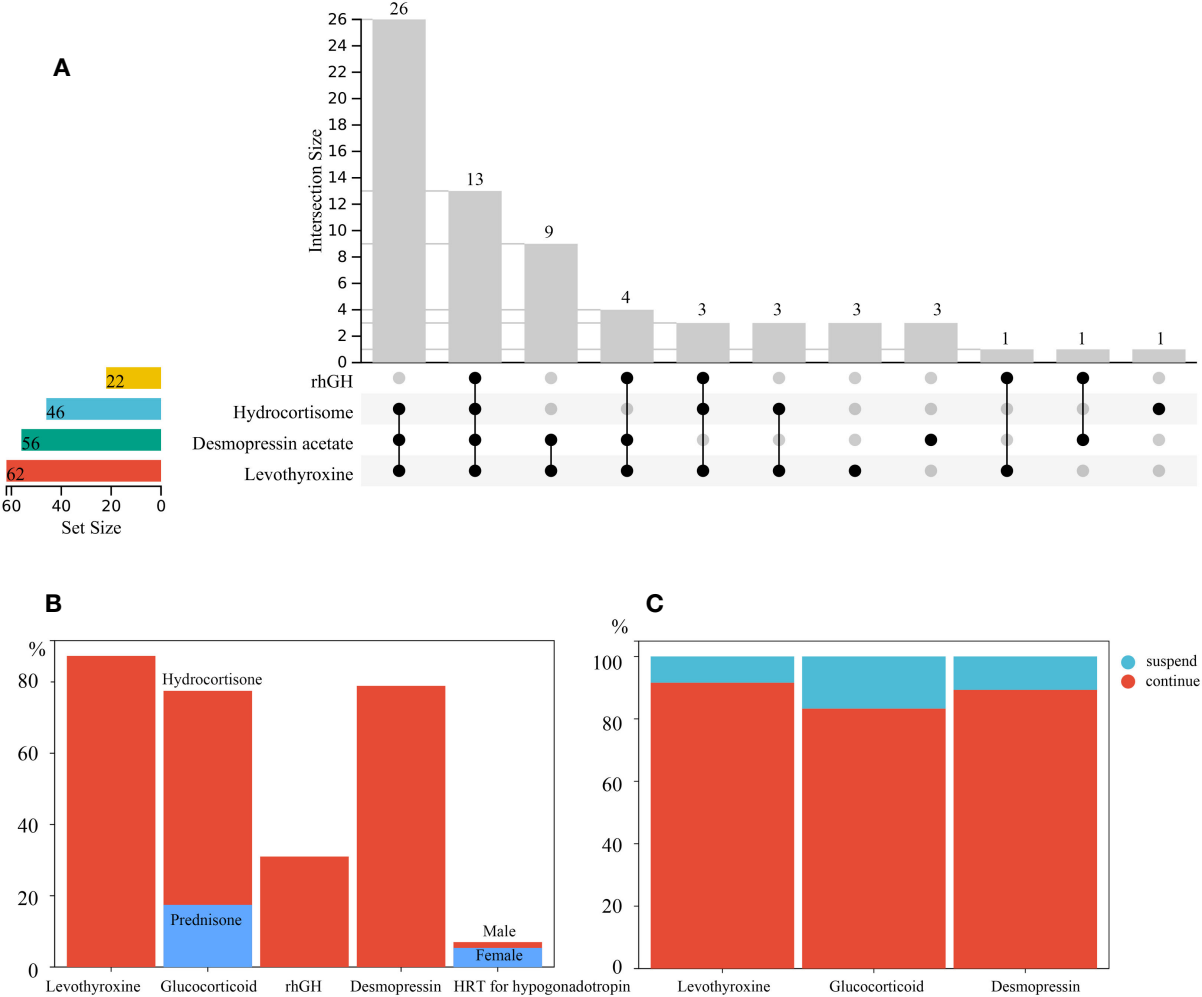
**(A)** Specific combinations of post operative hormone replacement therapy at follow-up. **(B)** Proportion of participants who were undergoing hormone replacement therapy at follow-up. The glucocorticoid used included hydrocortisone and prednisone. Male and female patients were colored differently for gonadotropin therapy; **(C)** Proportion of patients who were still on the same hormone replacement administered to them at discharge at follow-up.

Sixty-two patients (87.3%) were regularly reviewed in the endocrinology clinic. The major complaints included headache (5, 7.0%), visual impairment (24, 33.8%), polyuria (35, 49.3%), short stature (16, 28.1%), and obesity (31, 54.4%). At the time of follow-up, 4 patients (5.6%) did not require any form of hormone replacement. Most of the patients(94.4%) required at least one kind of hormone substitution treatment, while 74.7% patients were taking three or more. The specific combinations of hormone replacement therapies, except therapies for gonadotropin deficiency treatment, are shown in **Figure 3A**. There were 39 (54.9%) patients who required a combination of levothyroxine, desmopressin, and hydrocortisone replacement therapies. Among them, 13 (18.3%) also underwent growth hormone therapy. Further details of the hormone replacement therapy are shown in **Table 4** and **Figure 3B**.

**Table 4:** Hormone replacement therapy.

		Case	Total Cases	N%
Levothyroxine		62	71	87.3%
Glucocorticoid		55		77.5%
	Hydrocortisone	46		64.8%
	Prednisone	12		16.9%
rhGH		22		31.0%
Desmopressin		56		78.9%
Puberty Induce Therapy		5		
	Female	1	25	4.0%
	Male	4	46	8.7%

rhGH, recombinant human growth hormone.

Sixty-two patients (87.3%) were treated with thyroid hormone replacement therapy during the follow-up period. Of the 48 patients who developed hypothyroidism 1 week after the procedure, 44 (91.6%) still required oral levothyroxine replacement at the time of follow-up (**Figure 3C**). A total of 55 (77.5%) patients required glucocorticoid replacement therapy at the time of follow-up,46 (64.8%) of which received oral hydrocortisone. Oral prednisone was used as a long-term substitution in 12 (16.9%) patients. Of the 48 patients who had short-term postoperative adrenocortical insufficiency, 40 (83.3%) required maintenance of long-term oral glucocorticoid administration (**Figure 3C**).

A total of 22 patients (31.0%) had been treated with growth hormone, with a median duration of 19.6 (11.6, 27.6) months post operation. The shortest interval between tumor removal and the initiation of rhGH therapy was 3 months. Among the patients who had undergone rhGH therapy, four (18.2%) suffered tumor recurrence subsequently. Fourteen (29.8%) patients who were not receiving rhGH treatment had tumor recurrence. No statistically significant difference was found between the two groups (P=0.31).

In adolescent participants, 5 (29.4%) had started treatments to induce puberty onset, of which 3 (17.6%) met the age standard for delayed puberty (girls >13 years old and boys >14 years old without any puberty onset). Even though the other two male patients were younger than 14 years old, they were also started induction therapy. A total of 56 patients (78.9%) required oral desmopressin to control urine output at follow-up, of which 28 (50%) had unsatisfactory control of polyuria. Of the patients who required regular post-operative oral desmopressin replacement at discharge, 25 (89.3%) continued oral desmopressin replacement from the time of discharge until follow-up (**Figure 3C**). Only a few patients had temporary central diabetes insipidus after surgery.

## 4 Discussion

This is the first systematic research of postoperative endocrine function in Chinese patients with childhood-onset CP. The proportion of postoperative CP patients with short stature and hypothalamic obesity was seemingly high, and often require multiple long-term postoperative endocrine hormone replacement therapy. The results of this study provide evidence for long-term, individualized, and reasonable endocrine management in patients with childhood-onset CP.

### 4.1 Occurrence, clinical manifestations, treatment, and post-operative tumor recurrence in patients with childhood-onset CP

Previous studies have reported the population incidence range of CPs as 1.3 to 1.7 per 10^6^ patients, with approximately 30–50% of the cases being childhood-onset CPs (9–11). In this study, the age of patients ranged from 3 to 11 years old, with the highest proportion of patients being in the 3–5 years old group and the lowest being adolescents above 15 years old, which was consistent with prior reports (12).

The overall recurrence rate of CP is 19.8%–30.2% in patients after radical resection surgery (13–15). Many studies have explored the influencing factors of tumor recurrence, among which the complete removal of tumor tissue often relates to a lower tumor recurrence rate (13, 15). Our study showed that tumor recurrence was more common in patients who had undergone the endoscopic endonasal transsphenoidal approach than those who had underwent craniotomy. This may be related to the differing degree of tumor resection between the two approaches. Radical surgical resection of CPs, which may have the advantage of a fully exposed tumor for better surgical removal, was still the major surgical approach in our study. Most studies have reported no significant difference in tumor recurrence rate between patients who have undergone total tumor resection and partial tumor resection combined with radiotherapy (16, 17). However, the incidence of long-term endocrine hormone deficiency after partial tumor resection combined with adjuvant radiotherapy under the premise of protecting the hypothalamic-pituitary axis is lower than that after radical tumor resection. Therefore, the trend of pursuing radical tumor resection has gradually changed in some clinical centers (18, 19). It has been reported that the rate of radical resection of CP in pediatric patients is approximately 81.93% in China, but there is a lack of case reports related to radiotherapy (20).

The effect of rhGH therapy on postoperative tumor recurrence in pediatric patients with CP is often discussed by clinicians. Current European guidelines recommend growth hormone replacement therapy 1 year after surgery (21). Similarly, Chinese neurosurgical guidelines recommend rhGH therapy 2 years after a surgery without recurrence. In this study, the tumor recurrence rate of children who received rhGH therapy was not significantly higher, which is consistent with the results of most previous studies (22). In some cases, tumor recurrence was identified more than 35 months after surgery, suggesting that we should closely monitor tumor recurrence in high-risk children before decidingon rhGH therapy, and, if necessary, delay hormone replacement therapy. In patients who have undergone partial tumor resection, the effect of rhGH therapy on tumor progression is currently not clear, so this treatment still needs to be carefully evaluated in patients who have undergone incomplete tumor removal (23, 24).

### 4.2 Postoperative hypothalamic-pituitary dysfunction

Hypopituitarism is one of the primary postoperative complications in patients with CP, and it seriously affects their long-term quality of life. Hypopituitarism is more common in patients with childhood-onset CP than in those with adulthood CP (25, 26). Among the pediatric population, the deficiency rate of GH, FSH/LH, ACTH, and TSH is approximately 47%–93%, 61%–91%, 43%–92%, and 50%–86%, respectively (13, 25, 27–31). The deficiency rate of three or more pituitary hormones is approximately 75–100% (13, 26, 32). The incidence of central diabetes insipidus is approximately 70–90% and even 100% in pediatric patients under 10 years old (13). In this study, more than half of the patients required a combination of three hormones over a long duration. A high proportion of patients who developed multiple pituitary hormone deficiencies 1 week after surgery required the same hormone replacement therapy during the long-term follow-up period. This further verified that postoperative hypopituitarism in patients with CP is usually permanent and requires long-term hormone replacement therapy. These observations illustrate the importance of long-term endocrine outpatient follow-up and regular monitoring of hormone levels. In addition, although hydrocortisone can be a better substitution in mimicing physiological cortisol circadian rhythms, oral prednisone as a replacement is also documented in some patients. We will keep encouraging patients to switch to a more rational glucocorticoid replacement therapy during subsequent follow-up.

At the time of follow-up, nearly one-third of the children met the diagnostic criteria for short stature, regardless of whether growth hormone therapy was initiated. Growth hormone axis dysfunction often occurs in combination with other anterior pituitary hypopituitarism. Due to the young age of the patients and the short follow-up time in this study, the proportion of patients who actually needed growth hormone replacement may be higher. The low rate of gonadotropin therapy in our study may have been due to the small number of adolescents in our study. Children with panhypopituitarism often have central hypogonadism accompanied by short stature, and these children often have a strong desire to increase their target height. Therefore, it is recommended to start these children with growth hormone if there is no evidence of tumor recurrence after clinical evaluation. Subsequently, puberty onset could be induced at approximately physiologic timing of puberty with consideration of their ideal height.

The pre- and postoperative obesity rates of the children in this study were high, reflecting the damage to the hypothalamus caused by the tumor and surgical resection. High BMI will increase the body’s burden and risk of cardiovascular and cerebrovascular diseases (33). Moreover, it adversely affects the quality of life, social adaptability, and mental health of children postoperatively. Adequate pituitary hormone replacement therapy and increasing their participation in sports are alternative methods to control postoperative obesity (34).

### 4.3 Limitations of this study

As a retrospective study, it was difficult to obtain the unified quantification of endocrine hormone levels and the exact laboratory examination results due to the dispersed locations of outpatient clinics. Consequently, it was challenging to accurately assess the effect of hormone replacement therapy in patients. In addition, data related to hypothalamic function, such as learning ability, mood management, and circadian rhythm in children, were not collected by the clinics at that time, partially because of a lack of awareness of these “latent” manifestations. We will continue to track the postoperative hypothalamic hypopituitarism status of patients with CP and establish a data platform for management to lay the foundation for implementing individualized endocrine hormone replacement programs.

## 5 Conclusion

This is the first systematic evaluation of postoperative endocrine function in Chinese patients with childhood CP. Nearly a quarter of patients experienced postoperative tumor recurrence. Survival rates during follow-up are relatively high, but about three-quarters of patients require long-term multiple hormone replacement therapy. It is important to understand the incidence of hypothalamic-pituitary axis dysfunction of childhood-onset CP patients, in order to provide individualized and precise hormone replacement therapy and improve long-term prognosis.

## Data availability statement

The raw data supporting the conclusions of this article will be made available by the authors, without undue reservation.

## Ethics statement

The studies involving humans were approved by Medical Ethics Committee of Beijing Children’s Hospital, Beijing, China. The studies were conducted in accordance with the local legislation and institutional requirements. Written informed consent for participation was not required from the participants or the participants’ legal guardians/next of kin in accordance with the national legislation and institutional requirements.

## Author contributions

The study idea and design were conceived by DW. Clinical data were collected and analyzed by KF, XP, SL and JC. YM and YW conducted follow-up and collected corresponding data. YM interpreted the results and was the major contributor in writing the manuscript. MG and JG provide access of clinical data. DW, YM and RB revised the manuscript. All authors participated in the writing of the paper and critical discussions, read, and approved the final manuscript.
